# How does emotional content influence visual word recognition? A meta-analysis of valence effects

**DOI:** 10.3758/s13423-024-02555-8

**Published:** 2024-09-19

**Authors:** Pilar Ferré, Alberto J. Sánchez-Carmona, Juan Haro, Rocío Calvillo-Torres, Jacobo Albert, José Antonio Hinojosa

**Affiliations:** 1 Departament de Psicologia and CRAMC, Universitat Rovira i Virgili, Tarragona, Spain; 2 Centro Neuromottiva, Madrid, Spain; 3 Departamento de Psicología Experimental, Procesos Cognitivos y Logopedia, Universidad Complutense de Madrid, Madrid, Spain; 4 Instituto Pluridisciplinar, Universidad Complutense de Madrid, Madrid, Spain; 5 Centro de Investigación Nebrija en Cognición (CINC), Universidad Nebrija, Madrid, Spain

**Keywords:** Bayesian multi-level meta-analysis, Visual word recognition, Positive valence, Negative valence, Lexical decision task

## Abstract

Research on the interplay between language and emotion has shown evidence that the affective content of words influences their recognition. However, the direction of the effects is not clear, as there are mixed findings regarding the role of positive and, especially, negative valence. We conducted a Bayesian multi-level meta-analysis to examine the role of valence in visual word recognition, focusing on the lexical decision task. The results revealed a facilitative effect of positive valence on lexical decision times. That is, positive words led to faster responses than both negative and neutral words. In contrast, negative valence did not have any effect, although the analysis of several moderator variables suggested that there might be a facilitative effect in some cases, specifically, when negative words elicit very strong and intense emotions. These results shed light on the complexities of emotional word processing. They also point to the need for psycholinguistic models to take affective information into account, and thus provide a complete view of visual word recognition.

**Supplementary Information:**

The online version contains supplementary material available at 10.3758/s13423-024-02555-8.

## Introduction

The last decades have witnessed a growing interest in the study of the relationship between cognition and emotion. A common approach has been to examine the effects of the emotional content of the stimuli on cognitive processing, and ample evidence has been obtained concerning effects on attention, memory, reasoning and language (see Dolcos et al., 2014, for a review). Researchers have employed a wide range of stimuli, including images (Peyk et al., 2008), movies (Bos et al., 2013), sounds (Baumgartner et al., 2006), faces (Vuilleumier, 2005) and gestures (Flaisch et al., 2011), among others. This meta-analysis focuses on the role played by emotional content in word processing, an area that has recently expanded, although there is still a lot that remains unclear (for reviews, see Citron, 2012; Hinojosa et al., 2020; Palazova, 2014).

Most research on the interplay between language and emotion has been conducted from a dimensional perspective, which defines the human affective experience in terms of continuous variations in a few dimensions: emotional valence, emotional arousal and dominance (Bradley & Lang, 1999). Accordingly, the emotional content of words has been characterised by these dimensions. Emotional valence refers to the hedonic quality of the emotional response evoked by a word, from very negative/unpleasant to very positive/pleasant. For instance, “death” is a highly negative word while “party” is a highly positive word. Emotional arousal, in turn, indicates the intensity of that emotional response, ranging from very relaxing to very activating. For example, “pillow” is a highly relaxing word while “war” is a highly activating word. Finally, dominance indicates the control experienced in relation to the situation denoted by the word, and ranges from very low control to complete control. In this way, “accident” is a word associated with low control while “knowledge” is associated with high control.

Valence and arousal seem to be more relevant than dominance. Many studies have examined their influence on word processing (see Hinojosa et al., 2020, for an overview). Most findings in this field have been obtained with the Lexical Decision Task (LDT; Rubenstein et al., 1970), a paradigm that has been used extensively in psycholinguistic research into visual word recognition. In the LDT, participants judge whether a string of letters is a word or not in a particular language, while their reaction times (RTs) and their accuracy are recorded. Although these two variables provide valuable information about word processing, RT is considered to be more reliable than accuracy (i.e., errors may have multiple causes, some of them not related to lexical access). Consequently, theoretical models on word processing have mostly relied on RT data (see Balota & Chumbley, 1984). Although LDT is a relatively shallow task that does not require either a comprehensive processing of linguistic stimuli or access to the word’s meaning, many lexical and semantic variables have been demonstrated to affect the participants’ performance (Aguasvivas et al., 2018; Azuma & van Orden, 1997; Grainger & Jacobs, 1996; Keuleers et al., 2012; Mandera et al., 2020; Pexman et al., 2008).

Although both emotional valence and arousal influence RTs in the LDT, valence has been found to exert a larger effect than arousal (Kousta et al., 2009; Kuperman et al., 2014). However, the precise impact of emotional valence on word processing in LDT studies remains far from conclusive. Some studies show a general effect of emotional content, where both positive (e.g., “party”) and negative (e.g., “death”) words are recognised faster than words without an affective connotation (i.e., neutral words like “pen”; Kousta et al., 2009; Schacht & Sommer, 2009; Yap & Seow, 2014; however, see Palazova et al., 2013, for the opposite pattern of results). Nonetheless, a large body of research shows differential effects for positive and negative words. Indeed, experimental evidence largely points to a processing advantage for positive words (i.e., shorter RTs) over neutral and negative words (e.g., Kuchinke et al., 2005; Rodríguez-Ferreiro & Davies, 2019; Siakaluk et al., 2016), although there is some evidence of null effects of positive content (e.g., Bayer et al., 2012). Conversely, the influence of negative valence is more complex to determine: some studies show that negative words have a facilitated processing in comparison to neutral words (Citron et al., 2013; Kuchinke et al., 2007; Vinson et al., 2014), whereas other studies report an interference or inhibition (i.e., larger RTs for negative words compared to neutral words, e.g., Estes & Adelman, 2008; Larsen et al., 2008; Yao et al., 2016). There is also evidence of no effects of negative content in processing (Kuchinke et al., 2005; Larsen et al., 2006). Due to these inconsistencies, the main purpose of this meta-analysis was to clarify the role of emotional valence in visual word recognition, focusing on the LDT. Theoretical models in this field have traditionally neglected the role of affective variables (see Norris, 2013, for an overview). This has led to partial accounts of visual word recognition. Clarifying the effects of valence is a necessary step towards a better understanding of this process, which can be reflected in more complete models.

It is important to mention that the lack of experimental control may have contributed to the mixed results in the field. A turning point in this regard was the work by Larsen et al. (2006). These authors conducted a meta-analysis of studies that used the emotional Stroop task^1^ to test whether the inhibition observed for negative words might be explained by inadequate experimental control. They examined the lexical properties of negative, positive and neutral words from 32 emotional Stroop studies, revealing that negative words were longer, less frequent, and had fewer lexical neighbours than neutral words. In addition, they analysed the effect of valence on the lexical decision times for these words, using data from the English Lexicon Project (Balota et al., 2007). The results showed an inhibition for negative words compared to neutral words when covariates were not considered. However, when word length, lexical frequency, and number of lexical neighbours were included in the analysis, the inhibitory effect associated with negative words disappeared.

The influential study by Larsen et al. (2006) significantly shaped the course of subsequent research. Since its publication, experimental control in lexical decision tasks that include emotional words has been strengthened (e.g., Citron et al., 2014; Kousta et al., 2009; Kuperman et al., 2014; Vinson et al., 2014). This is critical given that a series of variables have been shown to affect RTs in the LDT. Among these variables are word length (e.g., New et al., 2006), lexical frequency (e.g., Balota & Chumbley, 1984), number of neighbours (e.g., Pollatsek et al., 1999), age of acquisition (e.g., Cortese & Khanna, 2007), concreteness (e.g., Barber et al., 2013) and imageability (e.g., Balota et al., 2004). More importantly, a linear relationship has been observed between valence and several of these variables, showing that positive words are more concrete, more frequent and familiar, contextually richer, and acquired earlier in life than neutral and negative words (Warriner et al., 2013). Therefore, it is necessary to rigorously match the valence conditions in these variables to reach reliable conclusions about valence effects. Furthermore, the rise of lexical decision mega-studies (see Balota et al., 2012) has made it possible to examine the effect of valence on large RT datasets once the effect of other lexico-semantic variables has been considered, and thus provide a real estimate of the role of valence in word recognition (e.g., Estes & Adelman, 2008; Rodriguez-Ferreiro & Davies, 2019; Vinson et al., 2014).

In addition to the issue of experimental control, some variables have been shown to interact with valence. Emotional arousal is arguably the most relevant and widely studied variable. There is evidence of a complex interplay between valence and arousal, with high arousal facilitating the recognition of negative words and conversely inhibiting the recognition of positive words (e.g., Citron et al., 2014; Hoffman et al., 2009; Larsen et al., 2008; Vieitez et al., 2021). This interaction is consistent with the avoidance-approximation model proposed by Robinson et al. (2004). According to this model, stimuli characterised by either high arousal or negative valence tend to elicit withdrawal strategies, as they are perceived as dangerous or threatening. On the other hand, stimuli that have either low arousal or positive valence elicit approach strategies because they are interpreted as safe or appealing. Thus, valence and arousal can elicit either congruent combinations of strategies, such as approach-approach and avoidance-avoidance, or incongruent combinations, such as approach-avoidance. According to this view, words characterised by low arousal and negative valence (e.g., *mold*), or by high arousal and positive valence (e.g., *sex*), generate conflicting strategies that need to be resolved before the participant responds, which leads to slower RTs. In contrast, words with high arousal and negative valence (e.g., *rape*), or with low arousal and positive valence (e.g., *relax*), lead to congruent strategies and thus faster responses. It should be noted, however, that the finding that valence effects are modulated by arousal is not consistent across the research, as some studies did not find an interaction between valence and arousal (e.g., Kuperman et al., 2014; Vinson et al., 2014).

Research has also documented an interaction between lexical frequency and emotional valence (Kuchinke et al., 2007; Kuperman et al., 2014; Palazova et al., 2011; Scott et al., 2009; Sereno et al., 2015). Again, evidence from different studies is rather mixed. In this sense, Kuperman et al. (2014) found that valence had a larger effect on low-frequency words than on high-frequency words. In other studies, the interaction revealed a differential effect between positive and negative words depending on their frequency: for high-frequency words, positive valence facilitated the lexical decision with respect to neutral and negative valence, whereas when the frequency was low, both positive and negative words were recognised faster than neutral words. This pattern is consistent with a general facilitation for positive words, which appears to be independent of frequency (Kuchinke et al., 2007). In contrast, facilitation for negative words seems to depend more heavily on specific moderating variables (Scott et al., 2009).

Finally, several studies have reported an interaction between valence and concreteness (Palazova et al., 2013; Yao et al., 2016). This interaction suggests that the effect of valence is more pronounced for abstract words than for concrete words. For example, Palazova et al. (2013) found an inhibition for both positive and negative words compared to neutral words; however, this effect was only observed in abstract words. Similarly, Yao et al. (2016) obtained an interaction between valence and arousal for abstract words, but not for concrete ones. The modulatory role of concreteness in valence effects has been explained in terms of the greater preponderance of emotional content in abstract words than in concrete words (Vigliocco et al., 2009). Indeed, abstract words are more often associated with emotional states and tend to have more affective properties than concrete words (Altarriba et al., 1999; Barsalou & Wiemer-Hastings, 2005). It should be noted, however, that like in the case of arousal and lexical frequency, there are also inconsistent findings with respect to concreteness. For instance, Kanske and Kotz (2007) reported an interaction between valence and concreteness, but in the opposite direction to that found by Palazova et al. (2013). Similarly, Yao et al. (2016) showed that the valence effects are restricted to concrete words.

As outlined above, several key questions remain unanswered in this line of research. The primary objective of this study was to determine the effect of emotional valence in visual word recognition, as assessed by the LDT. We aimed to elucidate whether both positive and negative valence facilitate word recognition or whether facilitation is restricted to positive words. We also aimed to determine to what extent the effect of valence is modulated by several affective and lexico-semantic variables, considering prior findings that show an interaction (e.g., arousal, frequency and concreteness). We therefore carried out a Bayesian multi-level meta-analysis. The Bayesian approach is particularly useful for finding solutions in a hierarchical model where all parameters are estimated simultaneously. In addition, it stands out for its flexibility to adapt to situations of different complexity, appropriately incorporating the uncertainty associated with the estimates and facilitating the interpretation of the different sources of variation present in the data. The results shed light on the complexities of emotional word processing. Hence, they are relevant for theoretical models of visual word recognition, which have not traditionally considered affective variables.

## Method

### Search strategy

We searched PubMed, ProQuest, Psycinfo and Scopus for papers published up to February 2023 using the search terms “valence”, “affective valence”, “emotional words”, “emotion words” along with “lexical processing”, “lexical decision” and “affective processing”. These terms were combined in different ways to identify all the relevant studies.

### Study selection

**Fig. 1:**
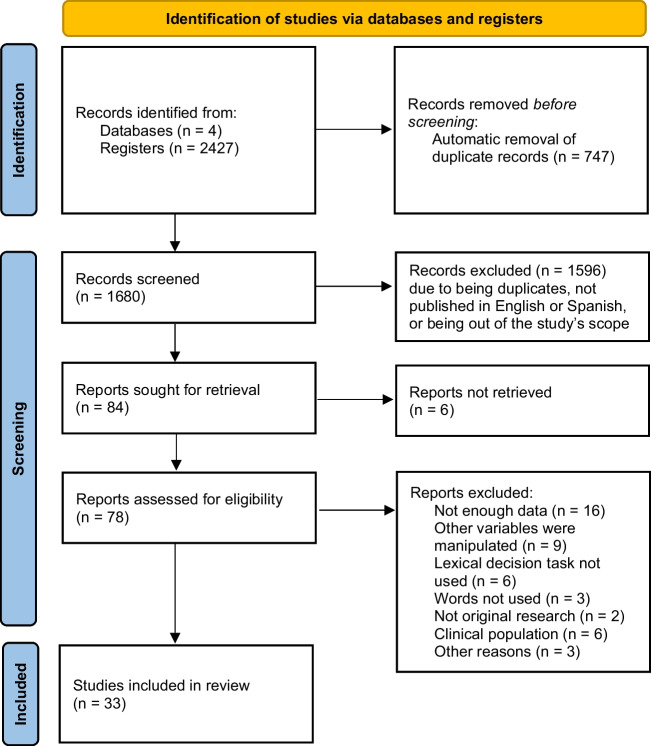
Flow diagram of the systematic review and meta-analysis. Figure adapted from the PRISMA 2020 statement (Page et al., 2021)

After the automatic elimination of duplicated studies, 1,596 studies were screened and a first inspection was conducted. This led us to discard duplicated studies not removed previously, as well as studies out of our scope and studies not written in English or Spanish. At this point, we had 84 reports to look at in detail. We then discarded records that did not report enough data, did not manipulate valence, did not employ the lexical decision task, did not use isolated words as experimental stimuli, or were not experimental studies. Therefore, our criteria for including papers in the meta‑analysis were that the research: (a) employed the lexical decision task, (b) was experimental research written in English or Spanish, (c) referred to non‑clinical populations, (d) reported valence means for the different groups of words according to their valence (positive/negative/neutral), and (e) reported RT data for the different groups of words according to their valence. In the end, 33 studies fulfilled the criteria and were included in the meta-analysis (see Fig. 1 for a flow diagram of the meta-analysis).

### Data extraction

The following information was extracted from each study: authors’ names, year of publication, sample size, means and standard deviations for the RTs, as well as means and standard deviations of the valence for the different groups of words (positive/negative/neutral). To explore the potential role of several psycholinguistic and affective variables as moderators of performance in lexical decisions about negative, positive and neutral words, we also retrieved the means and standard deviations whenever they were available for arousal, word frequency, length, imageability, age of acquisition, number of orthographic neighbours, and concreteness.

### Effect size calculation

The main goal of this meta-analysis was to examine the role of valence in word recognition. To this end, a separate meta-analysis model was computed for each possible comparison between the valence levels (positive vs. neutral, negative vs. neutral, and positive vs. negative). The lexical decision effect was operationalised as the difference in RTs between conditions. Therefore, a total of three meta-analysis models were adjusted to the data.

Within this framework, the same participants were tracked across the three valence conditions, so that the individual effect size of each study was computed under a repeated-measures design. Consequently, the mean and standard deviation of the lexical decision effect were extracted to compute the standardised mean change effect size (Cohen’s d_z_, see formula 1):

1 $${d}_{z}=\frac{{\underline{x}}_{2}-{\underline{x}}_{1}}{{s}_{diff}}$$

where x_1_ was always the neutral condition, except in the positive–negative comparison, where x_1_ was the negative condition. S_diff_ was the standard deviation of the difference scores (see formula 2):

2 $${s}_{diff}=\sqrt{{s}_{1}^{2}+ {s}_{2}^{2}-2\times r{\times s}_{1}{\times s}_{2}}$$

where s_1_ and s_2_ refer to the standard deviation of each pair of valence conditions and r indicates the correlation between their means. If the standard error (SE) was reported in the study, it was converted to the standard deviation with the following equation: $SD=SE\sqrt{n}$. Unfortunately, no study reported the correlation measure, so it was set for all data at the intermediate value of 0.5. The sample variance effect size was calculated with the following formula:

3 $$Var\left[{d}_{z}\right]=\frac{1}{n}+\frac{{{d}_{z}}^{2}}{2n}$$

Finally, d_z_ was converted to Hedges’ g (see formula 4) to control for the overestimation bias that the first measure has in the computation of the absolute value of the population standardised mean difference (Borenstein et al., 2009):

4 $$Hedges^{\prime}g=\left(1-\frac{3}{4df-1} \right)\times {d}_{z}$$

### Moderator variables

We aimed to study the influence of the following predictors on the overall effect size: valence, arousal, frequency of use, word length, imageability, age of acquisition and concreteness. These predictors were selected based on the availability of data in the reviewed literature. This literature revealed a remarkable heterogeneity in the measurement scales used to evaluate the words for each of the predictors. Consequently, a linear transformation was carried out to rescale the data, by using the most common value ranges according to the literature review. Specifically, valence was rescaled to a scale ranging from -3 to + 3; arousal to a scale ranging from 1 to 5; and imageability, age of acquisition and concreteness to a scale ranging from 1 to 7. Finally, lexical frequency was always considered on a scale of total frequency/million.

### Statistical analysis

We used a multilevel meta-analysis to capture as much information as possible about the different moderators. While some studies did not provide mean ratings for the different moderator variables, other studies verified that the valence conditions examined were matched in those variables. Another subgroup of studies focused on one of these predictors and included it in the design as a factor (e.g., high vs. low frequency) to examine its interaction with valence. This last strategy resulted in the same study reporting several effect sizes for each valence comparison. Consequently, this approach introduced dependency between individual data, which was considered by integrating a third layer in the structure of the meta-analysis model. Thus, in addition to nesting participants in their corresponding studies (level 1), these effect sizes were also nested into clusters made up of individual studies (level 2). Finally, the effects of these clusters were aggregated to estimate the overall true effect size (level 3). This approach involves estimating two different sources of heterogeneity, one for level 2 (within-cluster heterogeneity) and one for level 3 (between cluster heterogeneity).

A Bayesian model was used to overcome the underestimation bias of the between-study variance associated with the classical estimators (DerSimonian and Laird or Restricted Maximum Likelihood; DerSimonian & Laird, 1986). This approach has been reported to better estimate both the variance and mean. Specifically, it explores the whole distribution of the parameter and does not produce boundary estimates when the true value of variance is positive. Consequently, it also restricts the possibility of making liberal estimates of the mean effect size (Williams et al., 2018). The Bayesian meta-analysis makes it possible to base the analysis on considering relevant previous evidence, which would be modelled in terms of a prior distribution. In this case, the absence of similar meta-analytic studies motivated us to select a weakly informative prior distribution (Williams et al., 2018). A Normal prior distribution was proposed [$\mu \sim \aleph (\text{0,1})$ for the mean effect size and a Half-Cauchy prior distribution was specified for the variance [$\tau \sim HC(\text{0,0.5})$]. To interpret the Bayesian models, the credible intervals (CrIs) that contain the true value of the parameter with a 95% probability are reported. Two methods were used to reject the null hypotheses. First, the level of credibility associated with each hypothesis is provided, understood as the percentage of the posterior distribution that is consistent with the hypothesis proposed. Thus, the null hypothesis is rejected if the high-density region (95%) of the posterior distribution does not comprise the value of interest. In addition, evidence ratios are provided for greater clarity. These ratios, commonly referred to as Bayes factors, quantify the evidence provided by the estimate in favour of the effect versus the alternative interpretation. An evidence ratio greater than three was used to reject the null hypothesis.

Before fitting the Bayesian models, an influence analysis based on Cook’s distance was performed to detect studies that were particularly influential on the overall effect size. Studies with a Cook’s distance greater than 1 should be discarded from the model (Griffin & Oswald, 2022).

The possible presence of publication bias was explored by visually examining the symmetry of the funnel plots. Moreover, the possible asymmetry was quantified by regressing individual effect sizes onto the individual standard errors. If the presence of publication bias was detected, the corresponding overall effect size estimate was adjusted using the robust Bayesian meta-analysis procedure (RoBMA; Bartoš et al., 2022). This method relies on Bayesian model averaging procedures to combine in a single solution the different solutions proposed to adjust the effect size (i.e., PET-PEESE and selection model adjustments). In this way, the limitations outlined for these proposals are solved, and at the same time overcoming the binary decision-making approach that characterizes the frequentist framework. Thus, we first reported the Bayes factor quantifying the relative evidence of publication bias, followed by the adjusted estimate of the corresponding effect size. To perform these analyses, the multilevel nature of the data in this study was specified. Note that the RoBMA R package does not work with Hedges' g effect sizes, so Cohen's d was chosen as the estimated effect size.

The I^2^ parameter was used to quantify the heterogeneity present in each meta-analysis model (Higgins & Thompson, 2002). This parameter indicates the amount of variation not attributable to sampling error. However, due to the multilevel nature of the proposed models, heterogeneity had to be divided into two parts: one part associated with the difference of the true effect sizes within clusters and another part associated with between-cluster variation. Following Higgins and Thompson (2002), I^2^ values of 25%, 50% and 75% were considered as low, moderate and high heterogeneity, respectively.

For the meta-regression analysis, first, we combined the predictors associated with each valence condition into a single factor (absolute value of the difference) to reduce the multicollinearity in the model. Despite this, there was still a large set of moderators available. Therefore, the meta-regression was at risk of overfitting, spurious results and/or non-convergence. To minimise these risks, we used the Bayesian Regularized Meta-Analysis (BRMA) to select relevant moderators by shrinking small regression coefficients towards zero with regularising (LASSO) priors (Van Lissa et al., 2023). As the authors of this method point out, BRMA has been shown to be superior to restricted maximum likelihood (RMA) in rejecting irrelevant predictors. However, BRMA has also been shown to be worse at identifying the true effects of relevant moderators. Thus, after adjusting BRMA models, each moderator was tested individually to compare its predictive power with the null model. Importantly, since the BRMA method does not allow for missing data, regression analyses were performed after the multivariate imputation was applied with the chained equation method (MICE).

We analysed all data using the metafor (version 3.4–0, Viechtbauer & Viechtbauer, 2015), brms (version 2.18.0, Bürkner, 2017), rstan (version 2.26.13, Stan Development Team, 2019;) pema (version 0.1.2, Van Lissa et al., 2023) and mice (version 3.15.0, van Buuren & Groothuis-Oudshoorn, 2011) packages of the statistical software program R (version 4.1.3, R Core Team, 2018). The estimation of the effect size adjusted for the presence of publication bias was performed using the RoBMA R package (Bartoš et al., 2022). These analyses required the use of R version 4.3.3 for compatibility reasons.

## Results

For the negative-neutral meta-analyses, we included a total of 30 individual effect sizes, corresponding to 21 studies, involving a total sample size of 769 participants (see Table 1). The positive–negative meta-analyses were based on 27 individual effect sizes, obtained from 19 individual studies, involving a total sample size of 707 participants (see Table 2). Finally, 27 individual effect sizes were meta-analysed regarding the positive-neutral comparison. These data came from 19 individual studies that included data from 707 participants (see Table 3). Often, the same studies provided data for several of the experimental contrasts, which have been separated here for methodological reasons. A total of 23 different studies were meta-analysed in the present work, involving a total sample of 823 participants. See Tables 1, 2 and 3, for detailed effect size information.

**Table 1:**
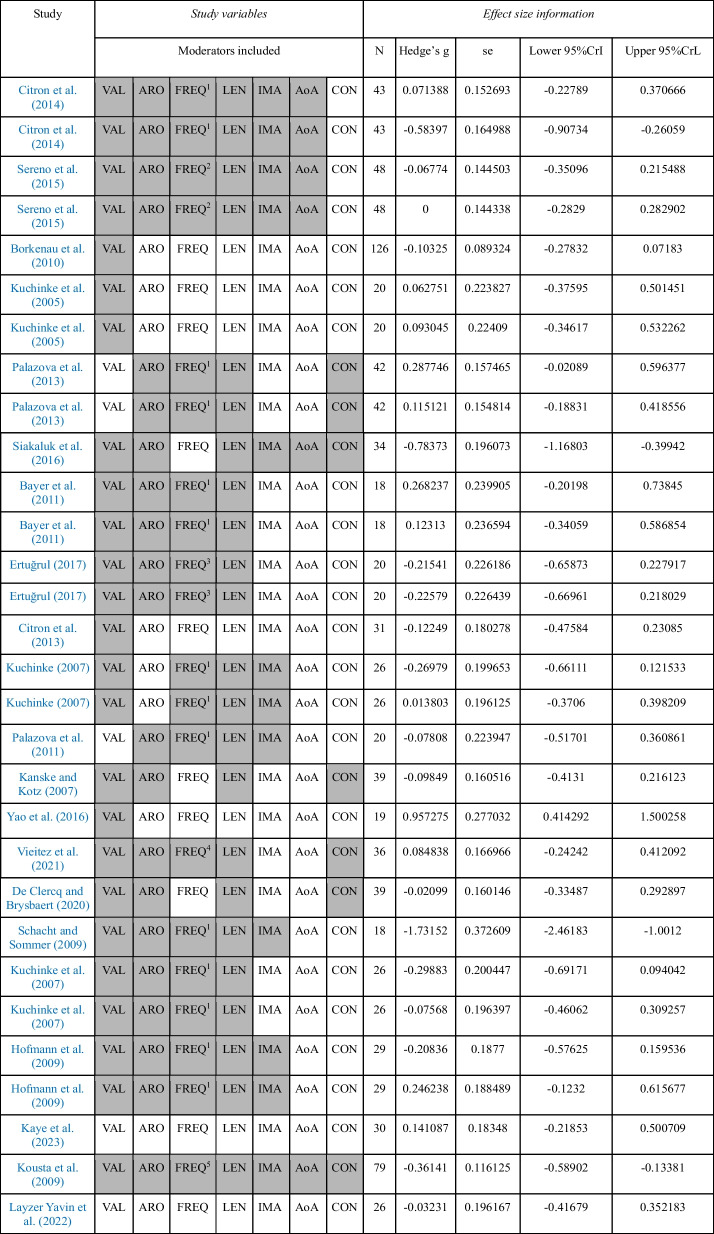
Studies included in the negative-neutral comparison

The moderators considered in each study are shadowed in grey. Percentage of missing data: VAL (16%), ARO (30%), FREQ (33%), LEN (23%), IMA (60%), AoA (80%), CON (76%)

Lexical frequency values were taken from: ^1^CELEX lexical database (Baayen et al., 1996), ^2^British National Corpus (BNC; Davies, 2004), ^3^BOUN Corpus (Sak et al., 2008), ^4^EsPal (Duchon et al., 2013), ^5^English Lexicon Project (ELP; Balota et al., 2007)

**Table 2:**
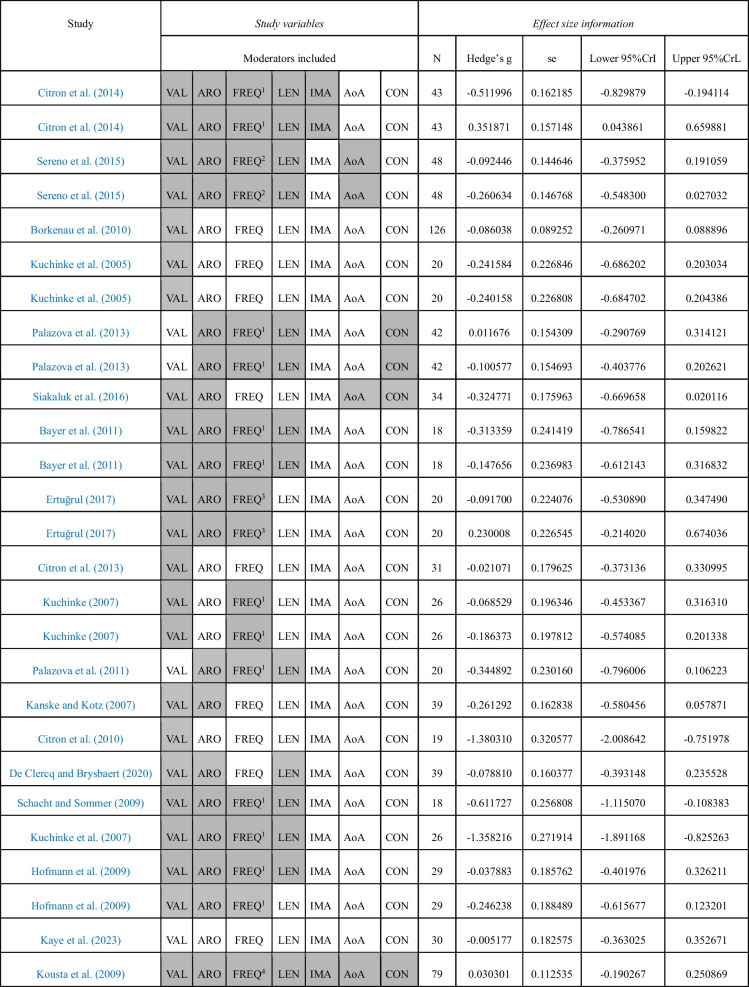
Studies included in the positive-negative comparison

The moderators considered in each study are shadowed in grey. Percentage of missing data: VAL (14.8%), ARO (29.6%), FREQ (33%), LEN (48.1%), IMA (88.9%), AoA (85.2%), CON (85.2%). Lexical frequency values were taken from: ^1^CELEX lexical database (Baayen et al., 1996), ^2^British National Corpus (BNC; Davies, 2004), ^3^BOUN Corpus (Sak et al., 2008), ^4^English Lexicon Project (ELP; Balota et al., 2007)

**Table 3:**
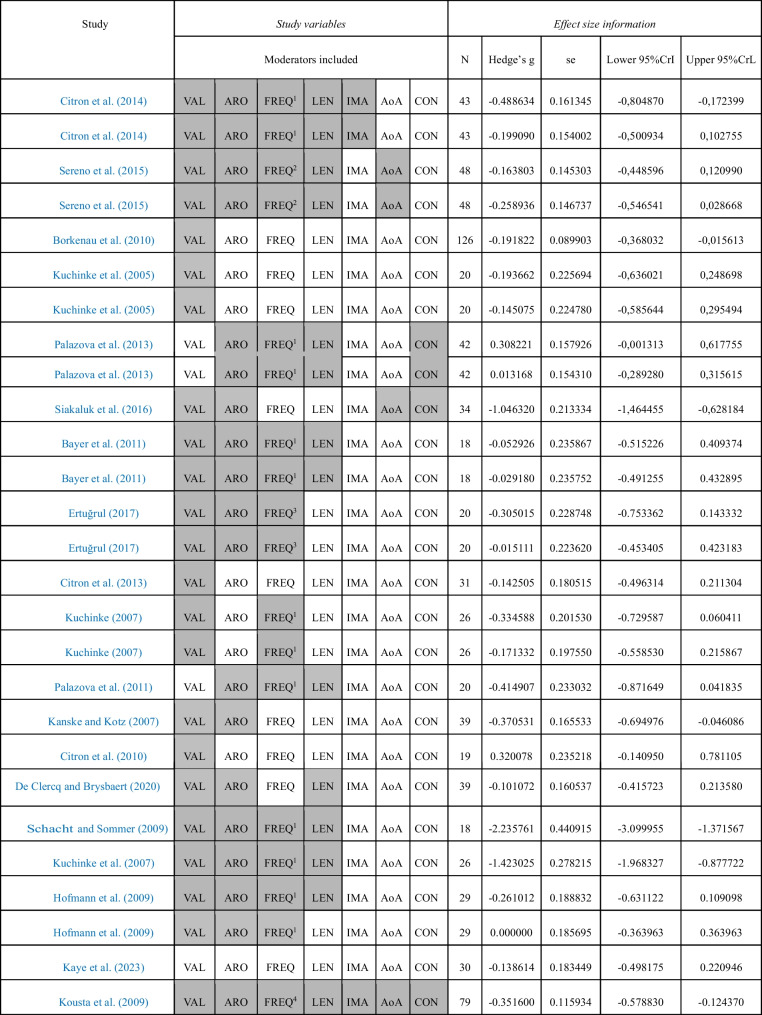
Studies included in the positive-neutral comparison

The moderators considered in each study are shadowed in grey. Percentage of missing data: VAL (14.8%), ARO (29.6%), FREQ (33.3%), LEN (48.1%), IMA (88.8%), AoA (85.1%), CON (85.1%). Lexical frequency values were taken from: ^1^CELEX lexical database (Baayen et al., 1996), ^2^British National Corpus (BNC; Davies, 2004), ^3^BOUN Corpus (Sak et al., 2008), ^4^English Lexicon Project (ELP; Balota et al., 2007)

### Meta-analysis

Influence analyses did not highlight the significant influence of any particular effect size on the overall effect sizes. Therefore, no study was discarded.

**Fig. 2:**
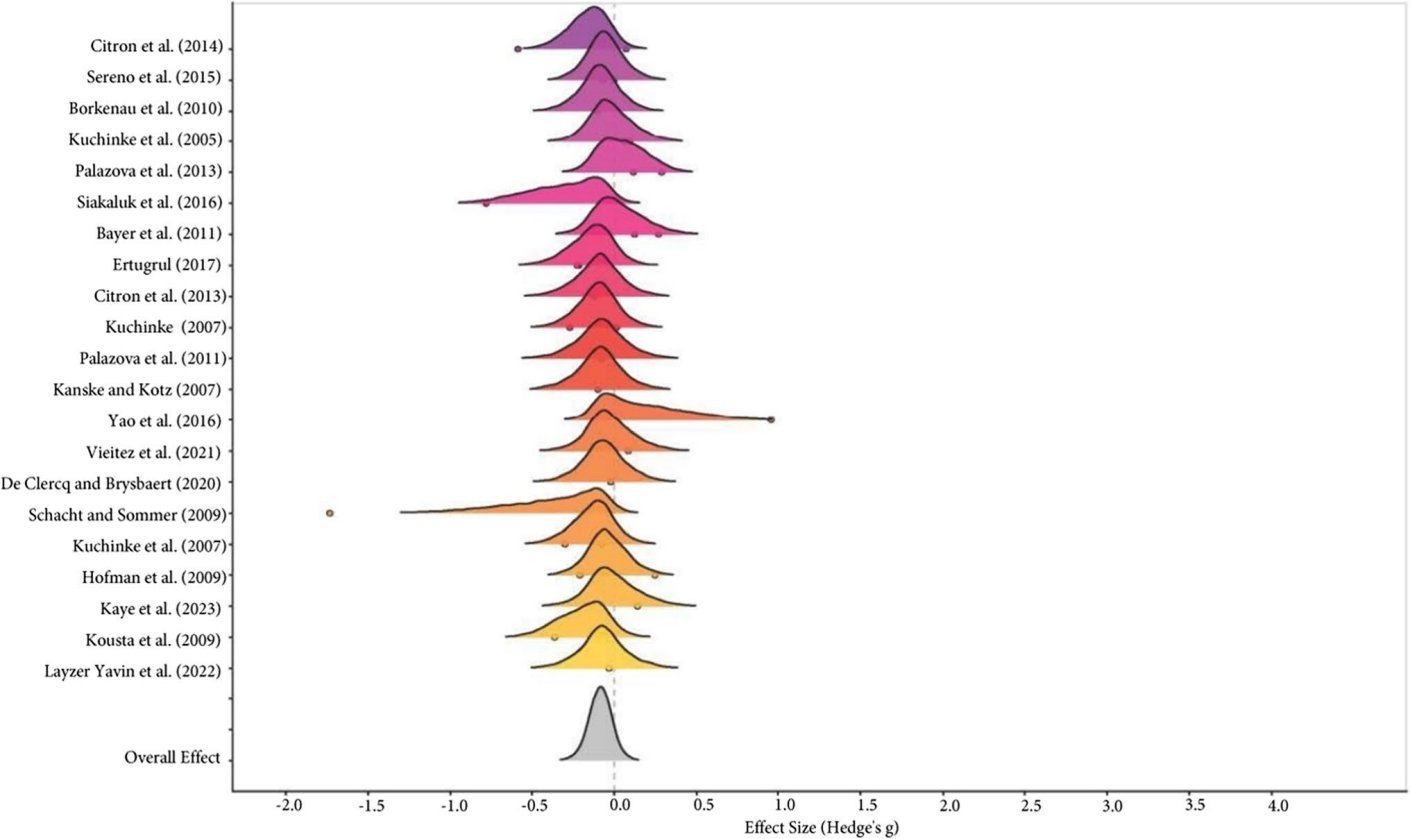
Forest plot for the negative-neutral meta-analysis

The comparison between negative and neutral valenced words did not show a significant difference in global effect size (Negative RT = Neutral RT: Hedges’g = -0.08, 95% CrI [-0.23, 0.06]), with moderate within-study ($\tau$ = 0.18, 95% CrI [0.01, 0.42]; I^2^_Level 2_ = 41.82%) and low between-study variability ($\tau$ =0.19, 95% CrI [0.02, 0.37]; I^2^_Level 3_ = 26%). In addition, 89% of the posterior probability function was consistent with this interpretation (credibility). Evidence ratio = 8.43 (see the forest plot in Fig. 2).

**Fig. 3:**
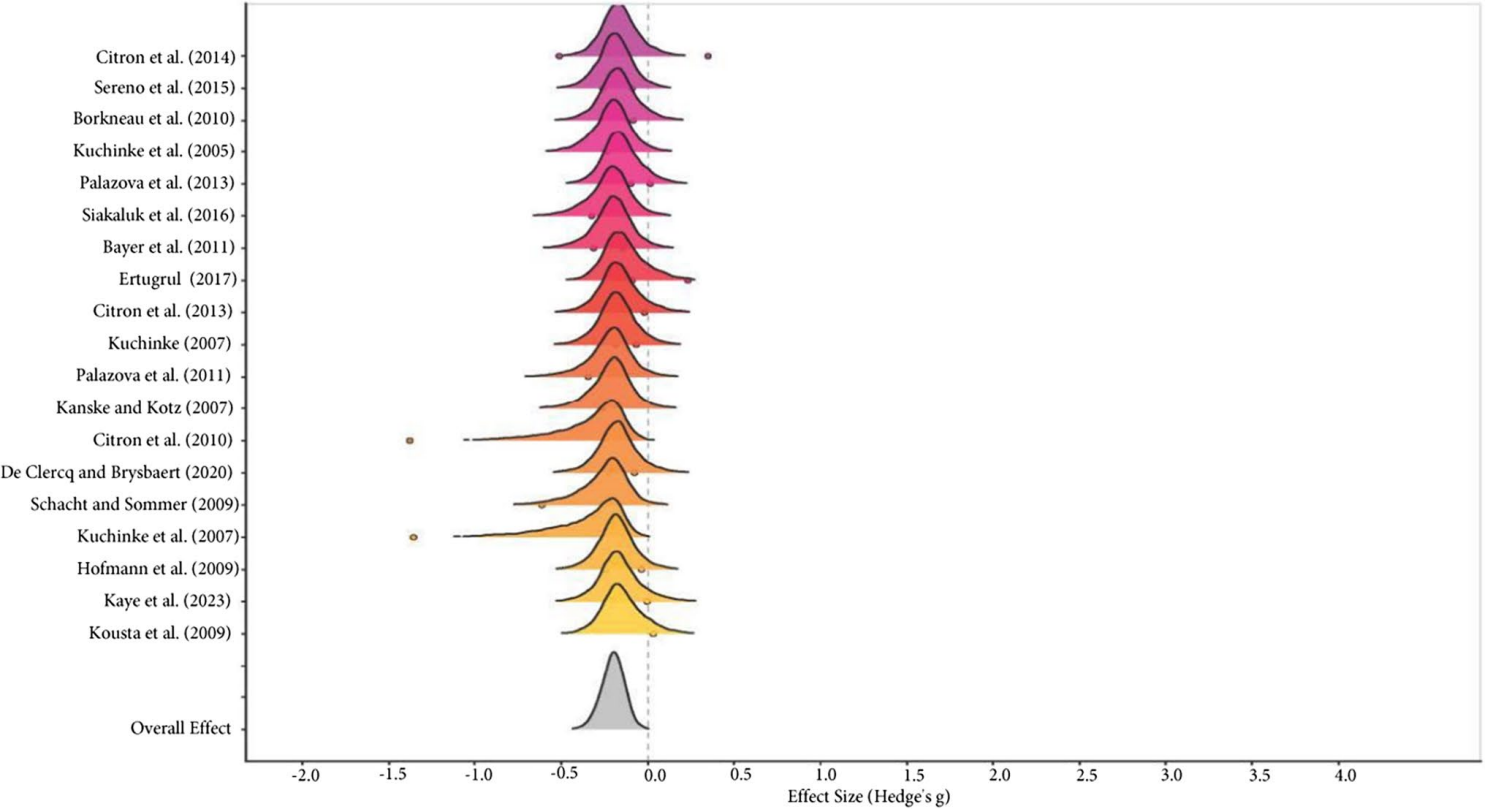
Forest plot for the positive–negative meta-analysis

The comparison between negative and positive words revealed a significant difference in global effect size (Positive RT < Negative RT: Hedges’g = -0.20, 95% CrI [-0.34, -0.07]), with high within-study ($\tau$ = 0.12, 95% CrI [0.01, 0.33]; I^2^_Level 2_ = 62.58%) and low between-study variability ($\tau$ = 0.21, 95% CrI [0.07, 0.37]; I^2^_Level 3_ = 0%). Furthermore, 100% of the posterior probability function was consistent with this interpretation (credibility). Evidence ratio = 525.32 (see the forest plot in Fig. 3).

**Fig. 4:**
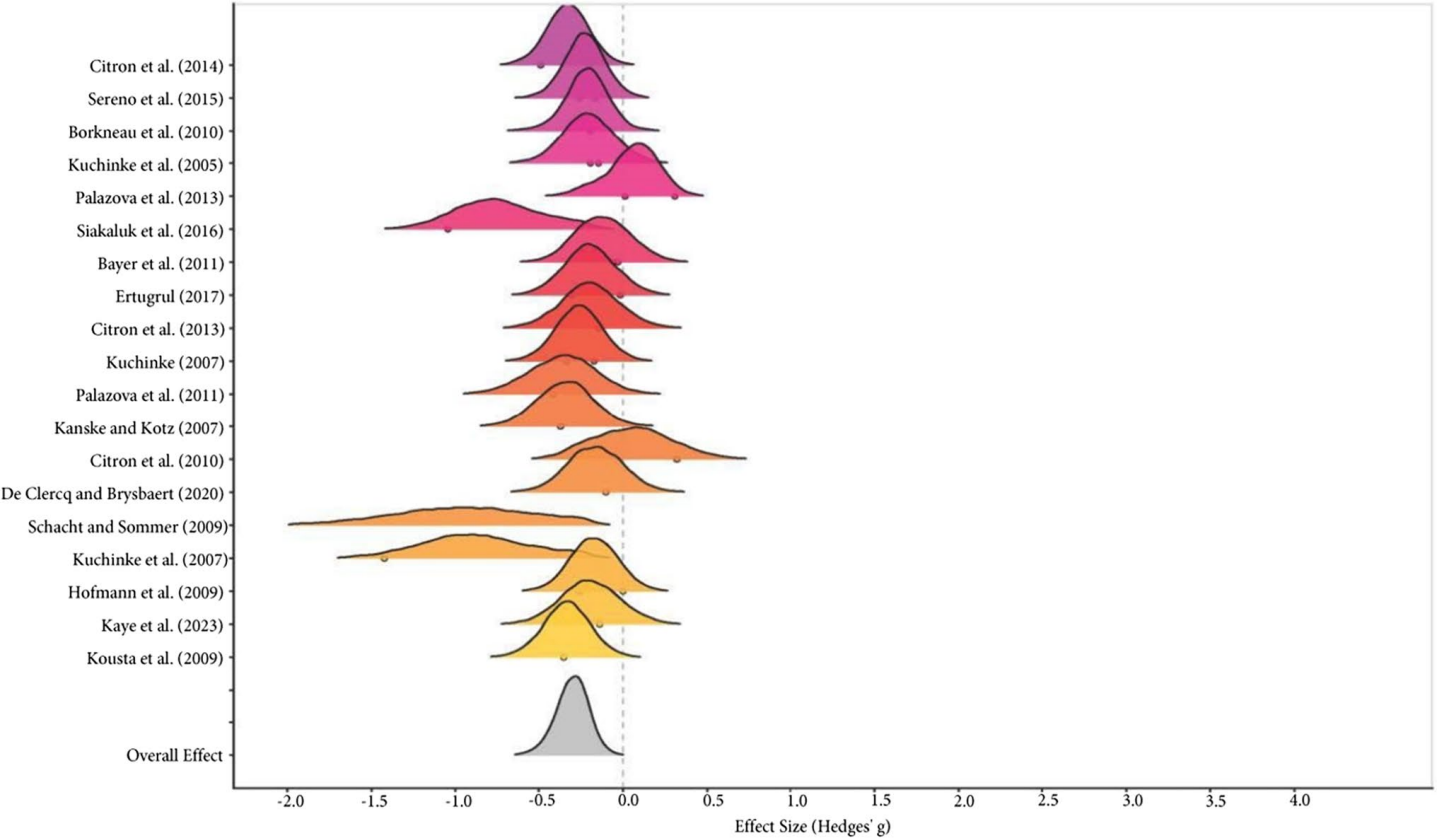
Forest plot for the positive-neutral meta-analysis

Finally, the comparison between the positive and neutral conditions also showed a significant difference in global effect size (Positive RT < Neutral RT: Hedges’ g = -0.30, 95% CrI [-0.51, -0.11]), with low within-study ($\tau$ = 0.34, 95% CrI [0.06, 0.61]; I^2^_Level 2_ = 0%) and high between-study variability ($\tau$ = 0.13, 95% CrI [0.01, 0.36]; I^2^_Level 3_ = 82.31%). In addition, 100% of the posterior probability function was consistent with this interpretation (credibility). Evidence ratio = 587.24 (see the forest plot in Fig. 4).

### Moderator analyses

The small number of available studies meant that several moderators could not be analysed (imageability, age of acquisition and concreteness, where imputation would have exceeded 75%). Consequently, it was only possible to study the association of effect sizes with the difference values of the following predictors: valence, arousal, frequency and length.

The negative-neutral meta-analysis model showed that a higher difference in valence values between conditions was negatively associated with global effect size (slope = -0.31, 95% CrI [-0.65, -0.00]). Considering that a negative global effect size indicates a faster RT for negative words compared to neutral words, this moderation effect suggests that the greater the difference in valence ratings between negative and neutral words, the faster participants in a lexical decision task will identify negative words. The same was true for the arousal predictor, that was negatively associated with global effect size (slope = -0.36, 95% CrI [0.12, 0.61]). This result indicates that the greater the difference between arousal ratings of negative and neutral words, the faster negative words will be identified. When all predictors were tested simultaneously with LASSO regularisation, no association was considered significant (see Online Supplementary Material (OSM) Tables 1 and 2 for the individual analysis and exploratory LASSO regularisation, respectively).

No predictor revealed significant results for the positive–negative comparison, neither in the joint analysis nor in the individual evaluation (see OSM Table 2). Finally, the global effect in the positive-neutral comparison was also not significantly associated with any predictor (in neither the joint nor the individual analyses) (see OSM Table 3).

### Publication bias

**Fig. 5:**
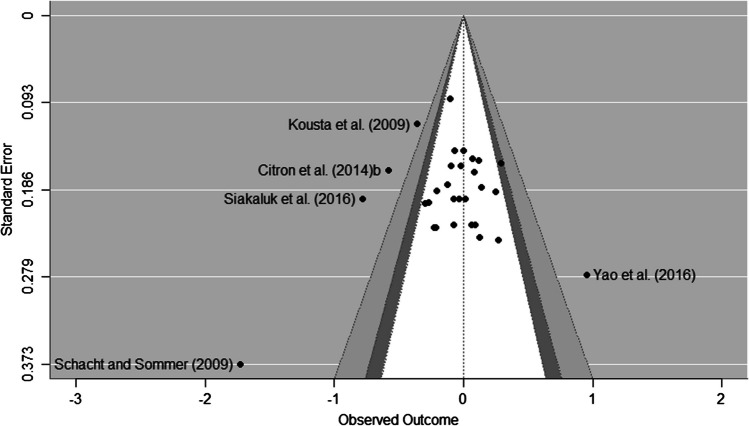
Funnel plot for the negative-neutral meta-analysis

**Fig. 6:**
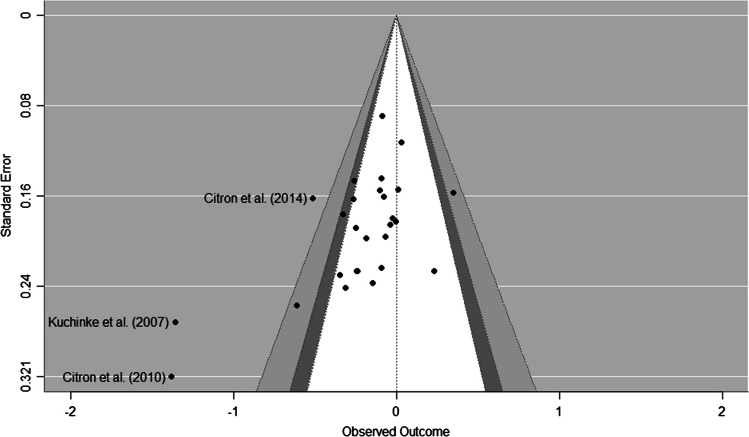
Funnel plot for the positive–negative meta-analysis

**Fig. 7:**
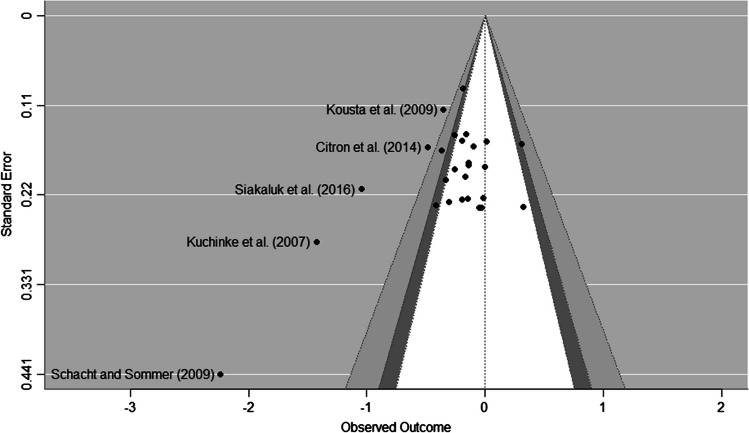
Funnel plot for the positive-neutral meta-analysis

Beyond the qualitative interpretation of the asymmetry of funnel plots (see Figs. 5, 6 and 7), the Egger’s regression test for the negative-neutral contrast was not considered significant. On the other hand, the analysis of publication bias with the RoBMA method showed greater evidence for the models indicating the absence of publication bias relative to those favouring a publication bias (Bayes factor = 0.7). However, the positive–negative meta-analysis suggests the presence of publication bias (b = -3.73, se = 1.18, 95% CrI [-6.19, -1.55, Credibility = 99%, Evidence Ratio = 90.74). Here, the RoBMA method quantified extreme evidence for the presence of publication bias (Bayes factor = 251.05). Consequently, the corrected effect size estimate was d = 0.017 [0.00, 0.225]. The same was true for the positive-neutral meta-analysis (b = -4.28, se = 1.44, 95% CrI [-7.26, -1.57], Credibility = 97%, Evidence Ratio = 32.17). In this case, the RoBMA analyses showed very high evidence of the presence of publication with a Bayes factor of 55.09. The adjusted effect size was d = 0.002 [-0.191–0.186].

## Discussion

The aim of this meta-analysis was to determine the effect of emotional valence on visual word recognition, focusing on data from the task most commonly used in this field, i.e., the lexical decision task. A secondary goal was to determine whether the potential effects of emotional valence are modulated by several lexico-semantic variables. The results showed a facilitative effect of positive valence on lexical decision times, and a lack of effect of negative valence. However, some effects for negative words emerged when valence difference and arousal were considered.

The results of the meta-analysis show a facilitated processing for positive words in comparison to both neutral words and negative words, although some caution is needed when interpreting this finding due to the existence of a publication bias. Indeed, the adjusted estimation computed to correct for this publication bias shows a clear decrease of the effect sizes involving positive words. Despite that, it is important to note that a processing advantage for positive words has been consistently found in mega-studies that have collected RTs to large sets of words. However, results from current mega-studies on the lexical processing of emotional words could not be included in the meta-analysis for methodological reasons (e.g., the absence of data to calculate Cohen’s d, or a linear rather than a categorical approach to valence). For instance, Kuperman et al. (2014) found a monotonic effect of valence on RT in a study involving more than 12,000 English words, retrieved from the English Lexicon Project (ELP; Balota et al., 2007). That is, lexical decision times decreased as valence values increased. The same pattern of results was reported in Spanish by Rodríguez-Ferreiro et al. (2019). Other studies have shown a categorical relationship between valence and RT, indicating that positive words are processed faster than negative words, regardless of valence extremity. This was the case in the study conducted by Estes and Adelman (2008), who examined the predictive power of valence values obtained from the Affective Norms for English Words (ANEW; Bradley & Lang, 1999) for lexical decision times from the ELP.

Several explanations have been provided for the positive valence advantage. One possibility is related to the positivity human bias in information processing (Walker et al., 2003), which also extends to language, with a preference for using positive words over negative ones (Augustine et al., 2011; Dodds et al., 2015). According to Kuperman et al. (2014), this bias may reduce the recognition threshold for positive words compared to neutral and negative words, leading to shorter RTs in lexical decision tasks. Another possible explanation of the facilitative effect is that positive emotional content is better elaborated and interconnected in the memory compared to negative or neutral content (Ashby et al., 1999; Isen et al., 1985). This may contribute to the higher semantic richness of positive words in comparison to negative and neutral words. Semantic richness can be defined as the amount and diversity of information that a word evokes (Kuperman et al., 2014). Several features have been proposed in the literature to contribute to the semantic richness of words, such as the number of associated words, semantic diversity, number of senses, imageability, and the degree of sensory experience evoked by a word, among others (see, for instance, Muraki et al., 2020). Kuperman et al. (2014) and Warriner et al. (2013) examined the correlation between valence and some of these variables, concluding that positive words are associated with higher semantic richness: they are more concrete, more imaginable, have a greater number of senses, and are associated with more sensory experiences. Importantly, there is considerable empirical evidence that semantic richness facilitates word processing (see Pexman, 2012, for a review). A proposed mechanism of this facilitation is the stronger feedback from semantics to orthography during processing (Pexman et al., 2008; Yap & Seow, 2014). Therefore, when a positive word is presented, its higher semantic richness (in comparison to negative and neutral words) would provoke a greater semantic activation, and consequently, stronger semantic-to-orthography feedback, resulting in faster word recognition. The lack of a moderator effect of lexical frequency on the positive valence effect is consistent with a semantic locus. If the locus of the valence effect was lexical, an interaction between valence and frequency should have been observed. Whatever the mechanism of the facilitative effect, either a decreased recognition threshold or a greater semantic richness, what is clear is that models of visual word recognition, in their present form, cannot account for this facilitative effect (see Norris, 2013). Therefore, they need to include affective information to provide a more complete picture of this process.

Regarding negative valence, this meta-analysis has revealed that it does not influence visual word recognition. Indeed, the comparison between negative and neutral words did not show a significant effect in global effect size, which agrees with the mixed findings in the field. The large-scale lexical decision studies reviewed above did not find consistent results, as they report both faster (Kousta et al., 2009; Vinson et al., 2014) and slower (Estes & Adelman, 2008; Kuperman et al., 2014; Rodriguez-Ferreiro et al., 2019) RTs for negative words with respect to neutral words. Researchers have tried to explain these two alternative patterns of findings within general models of emotional processing. In particular, the advantage in processing for emotional words, both positive and negative (e.g., Kousta et al., 2009; Vinson et al., 2014), aligns with the model of motivated attention and affective states (Lang et al., 1990, 1997). This model posits that emotional stimuli have a strong motivational relevance due to their critical role in self-preservation and protection. Consequently, both positive and negative words would attract more attention and be prioritised over non-emotional words, leading to faster RTs in the LDT. Alternatively, the automatic vigilance hypothesis (Pratto & John, 1991) has been proposed to explain the interference observed with negative words (Estes & Adelman, 2008; Kuperman et al., 2014; Rodriguez-Ferreiro et al., 2019). This hypothesis argues that, due to an evolutionary tendency to avoid threat and danger, humans have an innate predisposition to prioritise negative stimuli. This affects how much attention these stimuli receive and for how long our attention is captured (Estes & Verges, 2008). While the system takes steps to disengage from this attentional capture, the other cognitive tasks being performed in parallel are affected, such as deciding whether the stimulus is a word or not. The results of this meta-analysis do not support any of these proposals, but rather show the unreliable effects of negative valence.

Some authors consider that the contrasting findings regarding negative valence may be related to the stimuli included in the analyses. Kuperman et al. (2014) noted that large-scale studies that reported a facilitative effect for negative words (e.g., Kousta et al., 2009; Vinson et al., 2014) obtained their stimuli from ANEW (Bradley & Lang, 1999). However, considering that ANEW was explicitly developed to collect affective ratings, emotional words may be over-represented in this dataset. Interestingly, Kuperman et al. (2014) showed that the proportion of extremely negative words (i.e., with valence values below 2 on a 1–9 scale) was much larger in the ANEW sample than in the sample used in their own study. The analysis of the influence of several moderator variables performed here provides results in accordance with this. In particular, the comparison between negative and neutral words showed that the greater the difference in valence ratings between negative and neutral words, the faster participants responded to negative words. This suggests that extremely negative words may be processed differently from mildly negative words. Similarly, the RT to negative words decreased as the differences in arousal values between negative and neutral words increased (i.e., when negative words were much more arousing than neutral words). This last result aligns with the interaction between valence and arousal reported in several studies (e.g., Hoffman et al., 2009; Vieitez et al., 2021). According to the avoidance-approach model (Robinson et al., 2004), the processing of negative words with high arousal is facilitated because both high arousal and negative valence elicit congruent coping strategies (i.e., avoidance). In contrast, the opposite tendencies elicited by negative valence (avoidance) and low arousal (approach) may generate a conflict that results in slower RTs (Robinson et al., 2004). Further research is needed to directly address the possible differences in processing between more extreme and less extreme negative words, as well as the modulation by arousal. In any case, what these findings suggest is that positive and negative valence do not work in the same way: While the effect of negative valence is modulated by moderator variables (i.e., valence extremity and arousal), positive valence effects do not seem to depend on other properties of the words (at least, on the moderator variables examined here).

To conclude, the current meta-analysis provides evidence of the facilitative effect of positive valence in visual word recognition. However, the finding of publication bias suggests that data from LDT studies showing a positive valence advantage are more likely to be published than those reporting non-significant results. Besides some methodological considerations such as the need for publishing studies reporting null findings, our results indicate that it would be desirable to include unpublished results (e.g., from PsyArxiv) in future meta-analyses on this topic. Moreover, most mega-studies investigating the lexical processing of emotional words have examined the effects of valence linearly through linear mixed effects models. Data emanating from mega-studies relying in a categorical approach (i.e., negative vs. neutral vs. positive words) will shed light on the processing advantage for positive words. Regarding negative valence, it does not show any effect, although a facilitative effect might appear in very restricted circumstances, that is, when negative words elicit very strong and intense emotions. These findings show the subtleties of affective effects in visual word recognition and highlight that it is necessary for theoretical models to take affective information into account.

## Supplementary Information

Below is the link to the electronic supplementary material.

Supplementary file1 (DOCX 15 KB)

## Data Availability

Data can be found at: https://github.com/ascarmona91/EmotionLexicalDecision.git.

## Footnotes

1 The emotional Stroop task involves the unintended processing of the word for which participants are asked to name the colour of the ink it is written in, and therefore any variable that affects word processing should a priori affect the Stroop task (see Algom et al., 2004).

## Code availability

The scripts for the analyses can be found at https://github.com/ascarmona91/EmotionLexicalDecision.git.
