# Supplementary material for: How does emotional content influence visual word recognition? A meta-analysis of valence effects
Source: Psychon Bull Rev. 2024 Sep 19;32(2):570–87. doi: 10.3758/s13423-024-02555-8 (PMC12000208; doi:10.3758/s13423-024-02555-8)
Supplement: Supplementary file 1 — Supplementary file1 (DOCX 15 KB) [file 13423_2024_2555_MOESM1_ESM.docx]

Supplementary Table 1a. Negative-neutral meta-regression (individual analysis).

| **Moderator** | **slope [95%CrI]** | **Credibility** | **Evidence Ratio** |
| --- | --- | --- | --- |
| Valence | -0.31 [-0.59, -0.05] | 98% | 40.24 |
| Arousal | 0.36 [0.16, 0.57] | 100% | 688.66 |
| Frequency | 0 [0, 0.01] | 42% | 0.74 |
| Length | -0.21 [-0.63, 0.22] | 80% | 3.88 |

Supplementary Table 1b. Negative-neutral meta-regression (exploratory LASSO regularisation).

| **Moderator** | **Effect [95%CrI]** |
| --- | --- |
| Intercept | 0.28 [-0.24, -0.99] |
| Valence | -0.10 [-0.52, 0.21] |
| Arousal | 0.25 [-0.01, 0.55] |
| Frequency | 0.00 [-0.00, 0.01] |
| Length | 0.05 [-0.41, 0.63] |

Supplementary Table 2a. Positive-negative meta-regression (individual analysis).

| **Moderator** | **Slope [95% CrI]** | **Credibility** | **Evidence Ratio** |
| --- | --- | --- | --- |
| Valence | 0.1 [-0.05, 0.26] | 87% | 6.53 |
| Arousal | 0.07 [-0.08, 0.23] | 79% | 3.81 |
| Frequency | 0 [0, 0.01] | 76% | 3.14 |
| Length | 0.12 [-0.15, 0.39] | 77% | 3.4 |

Supplementary Table 2b. Positive-negative meta-regression (exploratory LASSO regularisation).

| **Moderator** | **Effect [95%CrI]** |
| --- | --- |
| Intercept | -0.41 [-1.28, 0.06] |
| Valence | 0.06 [-0.06, 0.28] |
| Arousal | 0.03 [-0.06, 0.18] |
| Frequency | 0.00 [-0.00, 0.01] |
| Length | -0.00 [-0.28, 0.27] |

Supplementary Table 3a. Positive-neutral meta-regression (individual analysis).

| **Moderator** | **Slope [CrI 95%]** | **Credibility** | **Evidence Ratio** |
| --- | --- | --- | --- |
| Valence | 0.05 [-0.48, 0.59] | 56% | 1.28 |
| Arousal | -0.08 [-0.23, 0.06] | 84% | 5.12 |
| Frequency | 0 [0, 0] | 51% | 1.05 |
| Length | 0.19 [-0.16, 0.55] | 82% | 4.55 |

Supplementary Table 3b. Positive-neutral meta-regression (exploratory LASSO regularisation).

| **Moderator** | **Effect [95%CrI]** |
| --- | --- |
| Intercept | -0.18 [--0.96, 0.75] |
| Valence | -0.03 [-0.57, 0.42] |
| Arousal | -0.03 [-0.19, 0.07] |
| Frequency | -0.00 [-0.00, 0.00] |
| Length | -0.03 [-0.43, 0.26] |
